# Optical Properties of GaN-Based Green Light-Emitting Diodes Influenced by Low-Temperature p-GaN Layer

**DOI:** 10.3390/nano11113134

**Published:** 2021-11-20

**Authors:** Jianfei Li, Duo Chen, Kuilong Li, Qiang Wang, Mengyao Shi, Dejie Diao, Chen Cheng, Changfu Li, Jiancai Leng

**Affiliations:** 1 Department of Physics, School of Electronic and Information Engineering, Qilu University of Technology (Shandong Academy of Science), Jinan 250353, China; likuilong123@126.com (K.L.); wangqiang@qlu.edu.cn (Q.W.); s19862127871@163.com (M.S.); diaodejie2021@163.com (D.D.); 2 International School for Optoelectronic Engineering, Qilu University of Technology (Shandong Academy of Science), Jinan 250353, China; mse_chend@ujn.edu.cn; 3 College of Physics and Electronics, Shandong Normal University, Jinan 250014, China; drccheng@sdnu.edu.cn; 4 School of Physics and Electronic Engineering, Taishan University, Taian 271000, China; sdtalcf1@163.com

**Keywords:** InGaN/GaN multiple quantum well, low-temperature p-GaN layer, photoluminescence, electroluminescence, localization effect

## Abstract

GaN-based green light-emitting diodes (LEDs) with different thicknesses of the low-temperature (LT) p-GaN layer between the last GaN barriers and p-AlGaN electron blocking layer were characterized by photoluminescence (PL) and electroluminescence (EL) spectroscopic methods in the temperature range of 6–300 K and injection current range of 0.01–350 mA. Based on the results, we suggest that a 20 nm-thick LT p-GaN layer can effectively prevent indium (In) re-evaporation, improve the quantum-confined Stark effect in the last quantum well (QW) of the active region, and finally reduce the efficiency droop by about 7%.

## 1. Introduction

Recently, GaN-based light-emitting diodes (LEDs), one of the most important optoelectronic devices, have attracted much attention owing to significant progress in related material growth and device manufacturing fields [1,2,3,4,5]. The external quantum efficiency (EQE) of high-performance GaN-based LEDs has been recorded to exceed 80%, and they have been extensively used in applications such as general illumination, displays, and communications [6,7]. However, InGaN-based LEDs emitting light of a longer wavelength in the green/yellow spectral range usually suffer from reduced emission efficiency, which is known as the green gap [8,9,10]. Since the first reports of green light-emitting InGaN LEDs, these devices have been used in a variety of commercial applications [11,12,13,14]. However, green LEDs suffer from a reduction in EL efficiency under high injection currents, which is known as “efficiency droop”. Generally, efficiency droop is caused by two main factors: (i) a high content of In can generate structural defects acting as nonradiative recombination centers due to a large discrepancy in atomic size between indium (In) and gallium (Ga), and a large lattice mismatch of 11% between InN and GaN; (ii) a large lattice mismatch between the InGaN well layer with a high In content and the GaN barrier layer causes strain-induced polarization, resulting in a strong quantum-confined Stark effect (QCSE) [15,16,17]. Previous studies showed that the introduction of a low-temperature (LT) p-GaN layer effectively improved the EL characteristics and reduced the efficiency droop in GaN-based blue LEDs [18,19,20,21]. Nevertheless, the effect of an LT p-GaN layer on longer-wavelength GaN-based LEDs has not been reported in detail, especially with different thicknesses of the LT p-GaN layer.

In this study, three GaN-based green LEDs with different thicknesses of the LT p-GaN layer were grown by metal–organic chemical vapor deposition (MOCVD), and their optical properties were studied using photoluminescence (PL) and electroluminescence (EL) spectroscopic methods. The PL was slightly affected by the LT p-GaN layer, while the EL showed a large difference in the peak energy, line width, and intensity at both 6 K and 300 K with the increasing thickness of the LT p-GaN layer. The underlying carrier dynamics in the GaN-based green LED were determined, as well as the temperature and current behavior of the EL peak energy and line width.

## 2. Materials and Methods

**Figure 1:**
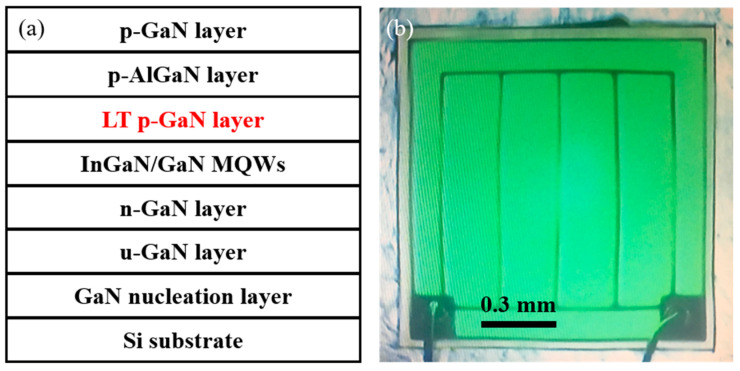
(**a**) Schematic diagram of green LED with a 20 nm-thick LT p-GaN layer. (**b**) Illuminated green LED chip at 300 K.

GaN-based green LEDs were grown on a trenched Si (111) substrate using MOCVD. The precursors of Al, Ga, In, N, and Si were trimethylaluminum (TMAl), trimethylgallium (TMGa), trimethylindium (TMIn), ammonia (NH_3_), and silane (SiH_4_), respectively. During the growth, first, a 25 nm-thick GaN nucleation layer was grown at 530 °C, followed by a 2 μm-thick undoped GaN layer, and a 2 μm-thick Si-doped GaN layer grown at 1080 °C. Four pairs of InGaN/GaN multiple quantum wells (MQWs) with 2 nm-thick InGaN wells and 14 nm-thick GaN barriers were grown under an ambient N_2_ atmosphere. For sample A, a 20 nm-thick Mg-doped p-AlGaN electron blocking layer (EBL) and a 150 nm-thick Mg-doped p-GaN contact layer were directly grown on the last GaN barrier layer of the MQWs at 1000 °C and 950 °C, respectively. However, for sample B (or C), before growing the EBL, a 20 (or 40) nm-thick LT p-GaN insertion layer was first deposited on the last GaN barrier layer of the MQWs at 820 °C. Figure 1a shows the structure with an LP p-GaN layer.

An LED chip with a size of about 1.16 × 1.16 mm^2^ was fabricated using a conventional mesa structure method, as shown in Figure 1b. The chip was mounted on a Cu cold stage in a temperature-variable, closed-cycle He cryostat (ARS, Macungie, PA, USA) to vary the sample temperature over a wide range of 6–300 K. The signals were analyzed using a Jobin-Yvon iHR320 monochromator (Horiba, Kyoto, Japan) equipped with a thermoelectrically cooled Synapse CCD detector. For the PL measurements, the 405 nm line of a semiconductor laser (CNI, Changchun, China) was used as the excitation light source. For the EL measurements, a Keithley 2400 source meter (Tektronix, Beaverton, OR, USA) was used as the excitation current source.

## 3. Results and Discussion

**Figure 2:**
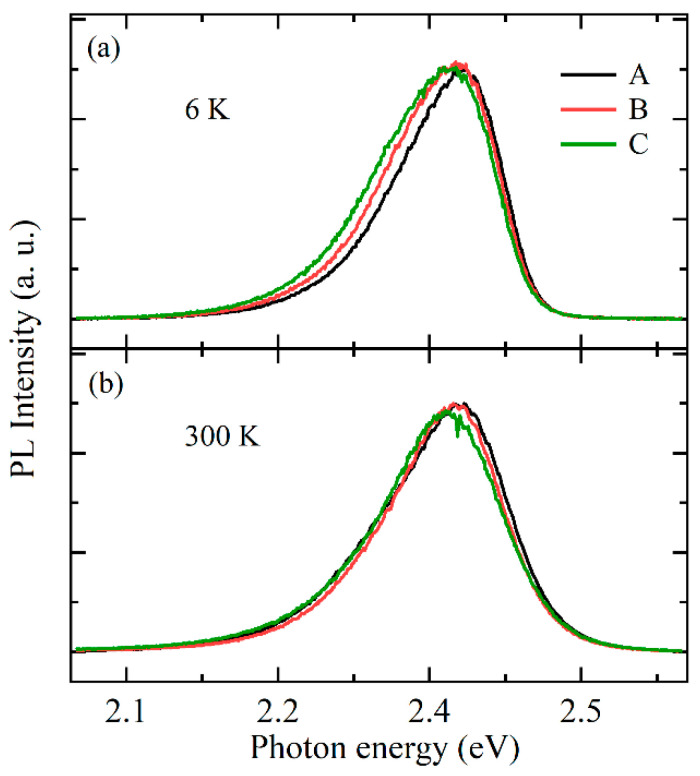
PL spectra of samples A, B, and C measured at 6 K (**a**) and 300 K (**b**).

Figure 2 shows the PL spectra of samples A, B, and C at 6 K and 300 K, which were dominated by a green emission, similar to that obtained from a green InGaN/GaN MQW-based LED [17,22,23]. For sample A, the peak energy and line width at 6 K, as shown in Figure 2a, were 2.432 eV and 122 meV, respectively, while those at 300 K, as shown in Figure 2b, were 2.428 eV and 144 meV, respectively. The variation in the PL peak energy and line width at different temperatures can be attributed to the conversion of carrier transfer mechanisms [23,24]. Compared with sample A, the peak energy, line width, and intensity of samples B and C slightly changed, as shown in Figure 2. This indicated that the LT p-GaN layer with different thicknesses slightly affected the PL characteristics of the GaN-based green LEDs.

**Figure 3:**
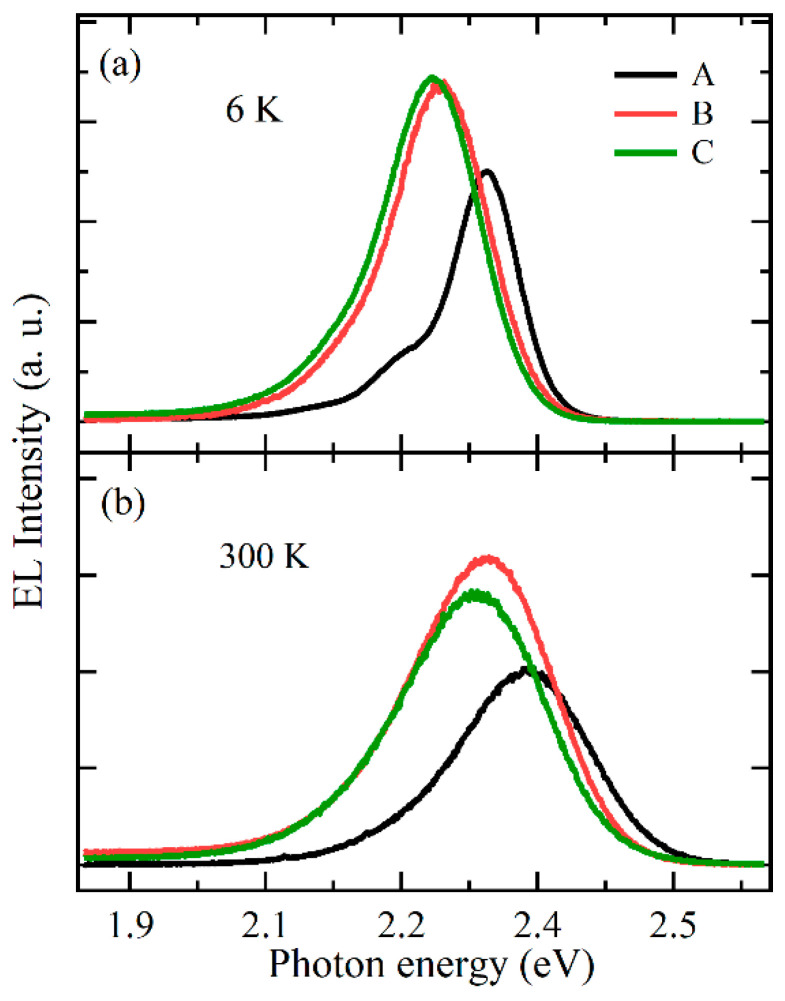
EL spectra of samples A, B, and C measured at 6 K (**a**) and 300 K (**b**) at 2 mA.

To further evaluate the details of the effect of the LT p-GaN layer on the emission mechanism of the active region of the MQWs, EL measurements were performed at 6 K and 300 K with different amounts of injected current. Figure 3a shows the EL spectra of three samples recorded at 6 K. For sample A, the peak energy and line width were 2.343 eV and 87 meV, respectively. With the increase in the thickness of the LT p-GaN layer from 0 to 20 nm at 6 K, the peak energy showed a significant red shift of about 49 meV, accompanied by an increase in the line width of about 40 meV. However, when the thickness of the LT p-GaN layer increased from 20 to 40 nm at the same temperature, the peak energy showed a slight red shift of about 10 meV, accompanied by an increase in the line width of about 4 meV. Figure 3a also shows that the integrated EL intensity significantly increased with the increase in the thickness of the LT p-GaN layer. The enhancement factor for the integrated EL intensity was about 1.74 for sample B and 1.79 for sample C. Moreover, with the increase in the thickness of the LT p-GaN layer at 300 K, as shown in Figure 3b, the peak energy and line width of the EL spectrum showed a similar trend; however, the integrated intensity first increased (1.66 for sample B) and then decreased (1.48 for sample C), unlike that at 6 K. The results indicate that the LT p-GaN layer significantly affected the EL characteristics and had a weak effect on the PL characteristics. These phenomena can be attributed to the differences between PL and EL, as described in a previous study: the PL spectra originated from every QW, while the EL spectra mainly originated from the last QW due to the difficulty of injecting holes from the p-type region into QWs [21,25,26]. Therefore, it can be inferred that the LT p-GaN layer had a greater effect on the last QW, rather than the other QWs far away from the LT p-GaN layer.

**Figure 4:**
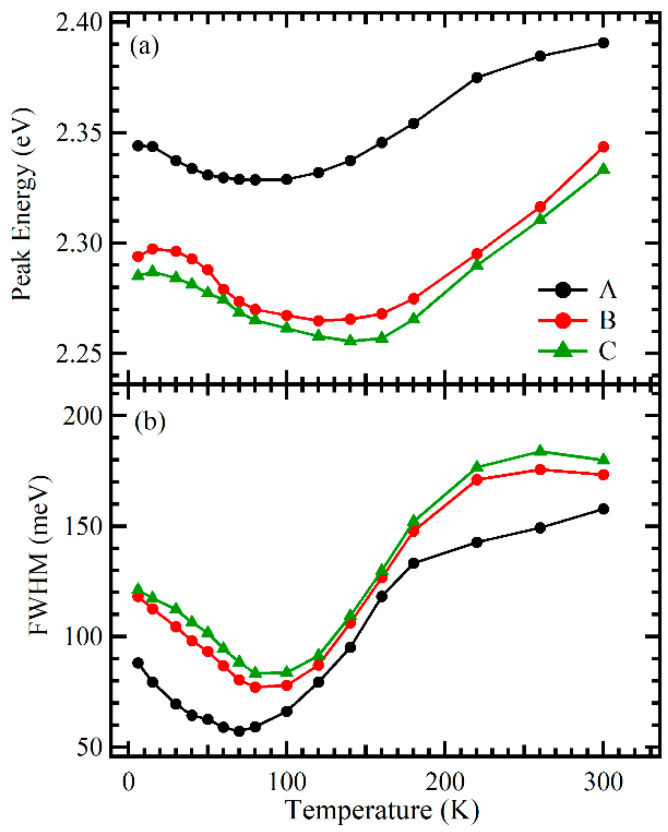
Temperature dependence of EL peak energy (**a**) and line width (**b**) of samples A, B, and C at 2 mA.

To further clarify the effect of the LT p-GaN layer on green InGaN/GaN MQWs, especially the last QW, Figure 4 shows the temperature dependence of the EL peak energy and line width of three samples at 2 mA. At 6 K for sample A, carriers exhibited a random distribution among the potential minima in the MQWs. When the temperature increased from 6 to 300 K, the temperature behavior of the peak energy showed an approximate V shape: the peak energy decreased as the temperature increased from 6 to 100 K, and the peak energy increased as the temperature increased from 100 to 300 K. At the same time, the line width decreased below the critical temperature of about 100 K, significantly increased from 100 to 180 K, and slightly increased from 180 to 300 K. These data indicate the following: as the temperature increased from 6 to 300 K, first, weakly localized carriers were thermally activated, relaxed down into other strongly localized states via hopping, and reached a saturated redistribution below about 100 K; then, the thermal broadening effect of localized carriers in the MQWs began to dominate the emission process, until most localized carriers became progressively mobile with a further increase in temperature to 300 K [16,23,27]. Moreover, the peak energy and line width of the three samples showed similar temperature-dependent behavior, as shown in Figure 4.

Furthermore, as the thickness of the LT p-GaN layer increased, the EL measurement results also exhibited the following properties. First, on increasing the thickness of the LT p-GaN layer from 0 to 20 nm, the EL spectra showed a red shift and spectrum broadening in the entire temperature range. This indicated that the active region in sample B had a higher In content than sample A (without an LT-GaN layer) because a higher In content in the active region could result in a smaller band gap energy and localized character of carrier recombination, owing to more significant composition fluctuations in the InGaN matrix. In other words, inserting an LT p-GaN layer between the active region of the MQWs and the p-AlGaN EBL could prevent IN re-evaporation from the active region of the MQWs (particularly in the last QW) during high-temperature p-AlGaN layer growth, leading to a high In content for sample B. Second, as the thickness of the LT p-GaN layer further increased from 20 to 40 nm, no significant change was observed in the peak energy and line width, indicating that inserting a 20 nm-thick LT p-GaN layer was sufficient for preventing the thermal degradation of the MQWs by suppressing the interdiffusion and re-evaporation of In in the MQW layers.

**Figure 5:**
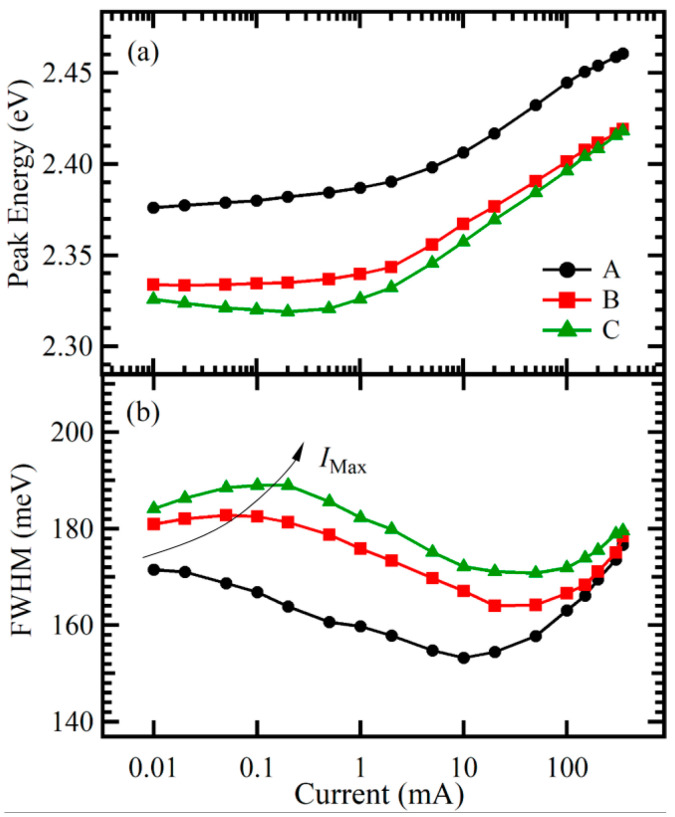
Current dependence of EL peak energy (**a**) and line width (**b**) of samples A, B, and C at 300 K. The critical currents *I*_Max_, corresponding to the initial minimum of the line width, are shown by an arrow as a guide to the eye.

In addition, Figure 5 shows the peak energy and line width of EL spectra of samples A, B, and C as a function of the injection current at 300 K. For sample A, the peak energy monotonically increased with the increasing injection current, while the line width first decreased in the low excitation range of 0.01–10 mA and then increased as the excitation power was further increased to 350 mA. These spectral behaviors indicate that the emission process of the MQWs was dominated first by the Coulomb screening of the QCSE and then by the filling of the localized states with the increasing injection current [17,28]. For sample B, however, different phenomena were observed. In contrast to sample A, the peak energy was almost unchanged, accompanied by the broadening of the line width with increasing excitation power from 0.01 to 0.05 mA, as shown in Figure 5. This shows that the defect-related nonradiative recombination started to affect the recombination process of the MQWs. With a further increase in the injection current, above 0.05 mA, since the nonradiative centers gradually became saturated, the Coulomb screening effect of free carriers started to dominate the recombination process of the MQWs [29]. Therefore, a similar phenomenon as discussed for sample A was observed in the higher current range of 0.01–350 mA of sample B. Moreover, as the thickness of the LT GaN layer continued to increase to 40 nm (sample C), the critical current *I*_Max_ increased, indicating an increase in the defect density. Moreover, as the thickness of the LT GaN layer increased, the decrease in the line width caused by the Coulomb screening of the QCSE became weaker. Thus, it can be concluded that the LT GaN layer could weaken the QCSE in MQWs, especially the last QW. The weakening of the QCSE can be attributed to a decrease in stress-induced piezoelectric polarization. Consequently, the LT GaN layer helped to reduce the intensity of the internal piezoelectric polarization field generated near the last QW due to the large lattice mismatch between the last barrier layer and the AlGaN EBL, thus improving the QCSE of samples B and C compared with sample A. Moreover, the EL peak energy decreased, and the line width increased with the increasing thickness of the LT GaN layer, as shown in Figure 3. This can be attributed to a competition between two factors: a higher In content resulted in a significant red shift and broadening of the EL peak; however, the improvement in the QCSE partially compensated for the red shift and broadening.

**Figure 6:**
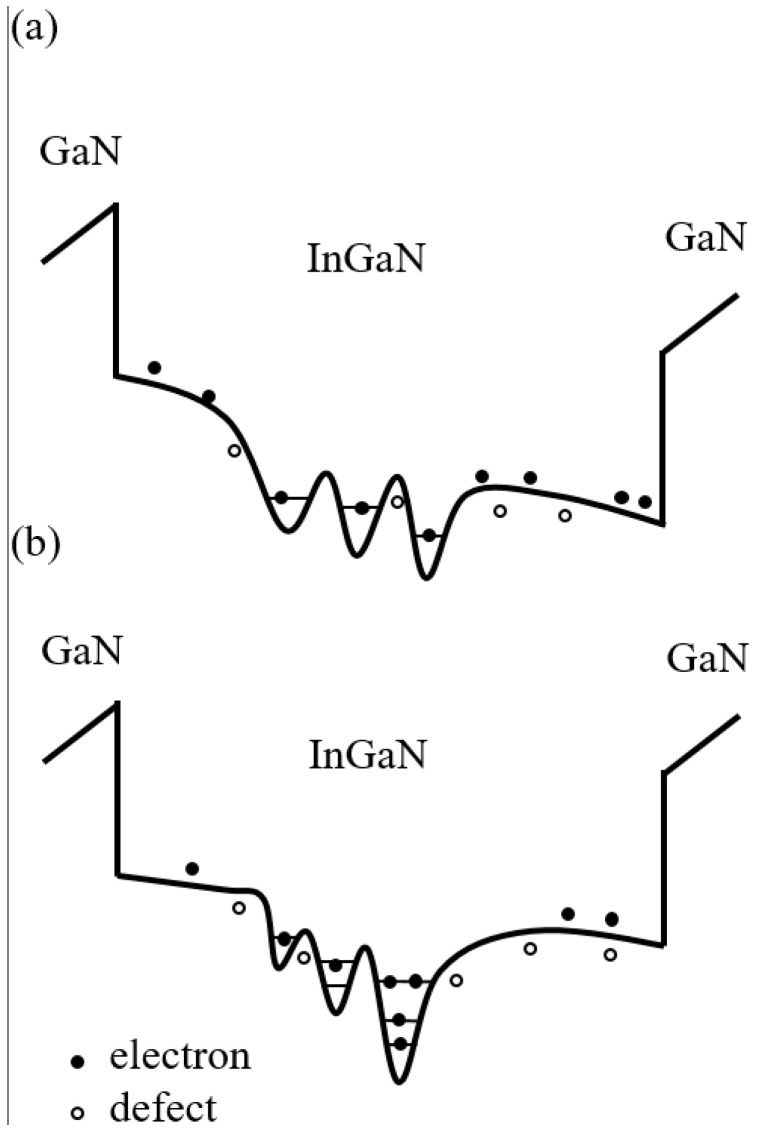
Schematic diagram of potential distribution in the last QW for samples A (**a**) and B (**b**), indicating the possible mechanism of carrier dynamics in the QW structure.

Considering the abovementioned EL characteristics, we found that the different thicknesses of the LT GaN layer could affect the In content, the QCSE, and the defect density in the InGaN-based green MQWs, especially the last QW. For sample A (without an LT GaN layer), the EBL was grown at 1050 °C after the last GaN barrier was grown. This caused the re-evaporation of In, reducing the In composition and making the In distribution more uniform in the active region of the MQWs. At the same time, the large lattice mismatch between the EBL and last barrier layer generated a strong internal piezoelectric polarization field near the last QW, increasing the major QCSE, as shown in Figure 6a. For sample B (with a 20 nm LT GaN layer), the active region should have had a higher In content and more significant potential inhomogeneity compared with sample A (without an LT-GaN layer) because inserting an LT p-GaN layer between the active region of the MQWs and the p-AlGaN EBL can effectively prevent In re-evaporation from the active region of the MQWs during high-temperature p-AlGaN growth. In addition, it should be noted that the LT p-GaN layer reduced the internal piezoelectric polarization field generated near the last QW, thus improving the QCSE of sample B. However, when the thickness of the LT GaN layer continued to increase to 40 nm, the In content and distribution, as well as the QCSE in the active region, were no longer affected. This was because the 20 nm-thick LT GaN layer was sufficient to prevent In re-evaporation and improve the QCSE according to the almost unchanged spectra of samples B and C. In addition, the growth temperature of the LT GaN layer was much lower than the general growth temperature of GaN, inevitably leading to an increase in the number of defects or dislocations, and even affecting the active area, i.e., the nonradiative recombination was enhanced, as shown in Figure 5.

**Figure 7:**
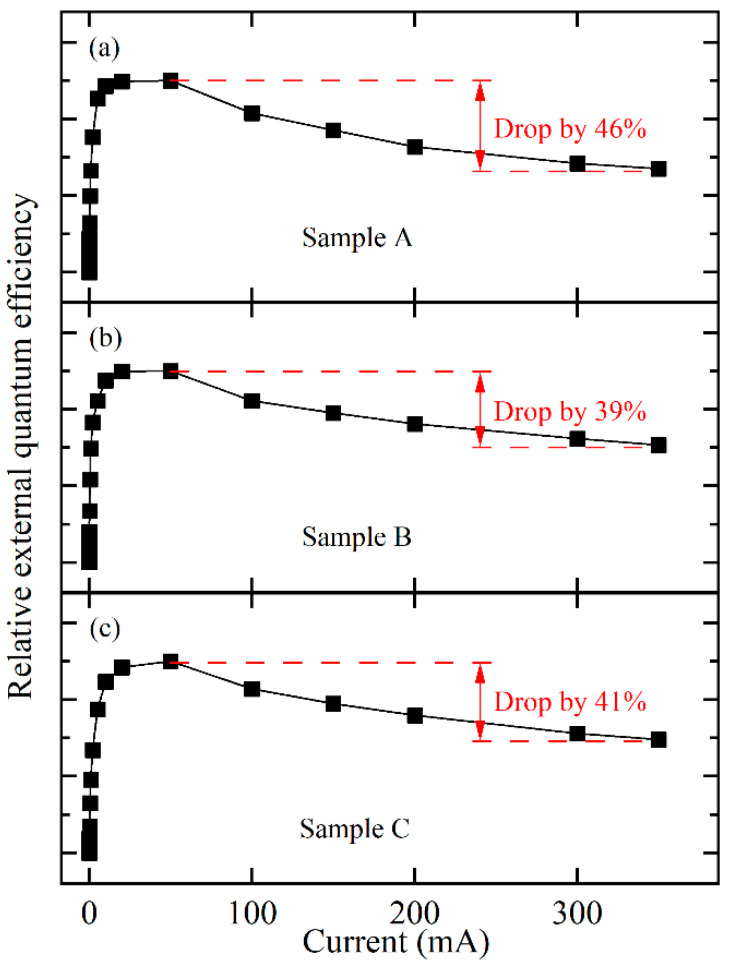
Relative efficiency of sample A (**a**), sample B (**b**), and sample C (**c**) as a function of injection current at 300 K.

To evaluate the effect of the LT p-GaN layer on the efficiency droop in these three samples, the integrated EL intensity divided by the current, i.e., relative EQE, was plotted as a function of the injection current at 300 K, as shown in Figure 7. As the injection current increased from 0.01 to 350 mA, these samples showed a significant efficiency droop: the EQE first increased due to the gradual saturation of nonradiative recombination centers and then decreased (i.e., efficiency droop), mainly due to electron leakage [19,20]. To qualitatively compare the efficiency droop for the three samples, efficiency droop can be defined as follows:

(1) $$F=\frac{\eta_{\mathrm{Max}}-\eta_{350\;\mathrm{mA}}}{\eta_{\mathrm{Max}}}$$

where *η*_Max_ is the maximum value of EQE from 0.01 to 350 mA; *η*_350 mA_ is the value of EQE at 350 mA.

As shown in Figure 7, when the thickness of the LT p-GaN layer increased from 0 to 20 nm, and even to 40 nm, the efficiency droop clearly decreased from 46% to 39% and then increased to 41%. Together with the previously observed enhancement of the EL intensity for sample B (Figure 3), this improvement in the efficiency droop for sample B can be mainly attributed to the increase in the hole injection efficiency and the suppression of electron leakage, owing to the enhanced localization effect by suppressing the evaporation of the In component, and the reduced QCSE by reducing stress-induced piezoelectric polarization. Furthermore, the increase in the efficiency droop for sample C can be attributed to the fact that the LT p-GaN layer was so thick that it produced defects and reduced the crystal quality of the p-AlGaN EBL and the p-GaN layer, even to the last QW, thus decreasing the hole injection efficiency.

## 4. Conclusions

In summary, we evaluated the effect of an LT p-GaN layer with different thicknesses on GaN-based green LEDs by EL and PL measurements in a temperature range of 6–300 K and an injection current range of 0.01–350 mA. The PL spectra were not affected by the LT p-GaN layer; however, the EL spectra showed a large difference in the peak energy, line width, and intensity at both 6 K and 300 K with the increasing thickness of the LT p-GaN layer. These results show that the In content of the active region of the MQWs significantly increased by preventing In re-evaporation from the active region of the MQWs and improving the QCSE after increasing the thickness of the LT GaN layer from 0 to 20 nm. Deterioration in the crystal quality occurred with a further increase in the thickness of the LT p-GaN layer from 20 to 40 nm, according to the temperature and current behavior of the EL peak energy and line width. In addition, the sample with a 20 nm-thick LT p-GaN layer showed the smallest efficiency droop over the entire injection current range tested at 300 K. The significant improvement in the optical performance can be mainly attributed to the increase in the hole injection efficiency and the suppression of electron leakage owing to the insertion of the LT p-GaN layer.

## Data Availability

The data that support the findings of this study are available from the corresponding author upon reasonable request.
